# Reflections on key methodological decisions in national burden of disease assessments

**DOI:** 10.1186/s13690-020-00519-7

**Published:** 2020-12-31

**Authors:** Elena von der Lippe, Brecht Devleesschauwer, Michelle Gourley, Juanita Haagsma, Henk Hilderink, Michael Porst, Annelene Wengler, Grant Wyper, Ian Grant

**Affiliations:** 1 Department of Epidemiology and Health Monitoring, Robert Koch Institute, Berlin, Germany; 2 Department of Epidemiology and Public Health, Sciensano, Brussels, Belgium; 3 Indigenous Data Analysis and Reporting Unit, Australian Institute of Health and Welfare, Canberra, Australia; 4 Department of Public Health, Erasmus MC University Medical Center, Rotterdam, The Netherlands; 5 Centre for Public Health Forecasting, National Institute for Public Health and the Environment, Bilthoven, The Netherlands; 6 Public Health Scotland, Edinburgh, Scotland, UK

**Keywords:** Burden of disease methodology, Disability-adjusted life years, Years of life lost, Years lived with disability, European burden of disease network, Population health measures, DALYs

## Abstract

**Background:**

Summary measures of population health are increasingly used in different public health reporting systems for setting priorities for health care and social service delivery and planning.

Disability-adjusted life years (DALYs) are one of the most commonly used health gap summary measures in the field of public health and have become the key metric for quantifying burden of disease (BoD).

BoD methodology is, however, complex and highly data demanding, requiring a substantial capacity to apply, which has led to major disparities across researchers and nations in their resources to perform themselves BoD studies and interpret the soundness of available estimates produced by the Global Burden of Disease Study.

**Methods:**

BoD researchers from the COST Action European Burden of Disease network reflect on the most important methodological choices to be made when estimating DALYs. The paper provides an overview of eleven methodological decisions and challenges drawing on the experiences of countries working with BoD methodology in their own national studies. Each of these steps are briefly described and, where appropriate, some examples are provided from different BoD studies across the world.

**Results:**

In this review article we have identified some of the key methodological choices and challenges that are important to understand when calculating BoD metrics. We have provided examples from different BoD studies that have developed their own strategies in data usage and implementation of statistical methods in the production of BoD estimates.

**Conclusions:**

With the increase in national BoD studies developing their own strategies in data usage and implementation of statistical methods in the production of BoD estimates, there is a pressing need for equitable capacity building on the one hand, and harmonization of methods on the other hand.

In response to these issues, several BoD networks have emerged in the European region that bring together expertise across different domains and professional backgrounds. An intensive exchange in the experience of the researchers in the different countries will enable the understanding of the methods and the interpretation of the results from the local authorities who can effectively integrate the BoD estimates in public health policies, intervention and prevention programs.

## Background

Summary measures of population health (SMPH) are gaining popularity in the recent years and are increasingly used in different public health reporting systems as an input for setting priorities for health care and social service delivery and planning.

SMPH can be broadly divided in two groups – indicators for health expectancies and health gaps [1]. A health expectancy indicates which part of the total life expectancy is spent in good health, and includes measures of healthy life expectancy [2]. A health gap quantifies the difference between the actual health of a population and some stated norm or goal for population health [1]. Each of these summary measures has a wide-range of subset measures that has been further developed and implemented in the last few decades.

Disability-adjusted life years (DALYs) are one of the most commonly used health gap summary measures in the field of public health and have become the key metric for quantifying burden of disease (BoD) [3–5]. The DALY metric quantifies the gap between a life lived in perfect health and the current health status, as the number of healthy life years lost due to illness (Years Lived with Disability, YLDs) and premature death (Years of Life Lost, YLLs). The DALY also allows for monitoring changes in the health of a given population and for comparing the health of different populations. BoD studies are becoming increasingly popular and their indicators are used as a means to influence national and local policy decisions. Driven by the Global Burden of Disease (GBD) projects initiated in the early 1990s, the DALY has become the dominant SMPH in BoD studies.

Since 2010 the Institute for Health Metrics and Evaluation (IHME) publishes regular updates of the GBD study for the entire world [6]. IHME is subsequently and continuously developing and improving their methodology and data recruitment for estimating the DALYs along with all the other main components. For 2019 GBD will report estimates on premature death and disability for more than 350 diseases and injuries (including more than 1200 disease sequelae) and will estimate the contribution of 87 risk factors to this disease burden [7]. The coverage of diseases and risk factors is a dynamic process and is extended with each of the study updates.

Driven by the impact of the GBD study, many countries across the world have adopted the BOD approach, producing independent DALY estimates or building on the work of the World Health Organization (WHO) and IHME [8–11]. Despite the increasing prominence of the BoD approach, several challenges remain. The BoD methodology is complex and highly data intensive, which has led to major disparities across researchers and nations in their capacity to perform BoD studies, to interpret the soundness of available BoD estimates, or to advocate for the use of BoD methods [12]. BoD as a generally standardized approach nonetheless requires different methodological choices, and lack of harmonization in these may hamper comparisons across studies.

In response to these needs, several countries and BoD researchers have set up ad hoc partnerships. In 2016, the WHO Regional Office for Europe (WHO-EURO) launched a European BoD network, aiming to intensify links between WHO, IHME and the WHO-EURO member states [13]. More recently the EU COST Action Burden of Disease network was established to serve as a technical platform to integrate and strengthen capacity in BoD assessment across Europe and beyond [12]. One of the key aims of the network is to provide a platform to support methodological insights and advances in performing national BoD studies.

## Aims

In this paper, experts from the COST Action Burden of Disease network reflect on some of the key methodlogical challenges and decisions in performing BoD studies. The content of this paper has been organized to reflect as far as possible the sequential steps which need to be followed to carry out a national BoD study (Table 1). The paper is not striving to describe all the calculation steps to estimate DALYs, but rather to outline some of the most important steps and decisions to be undertaken in a stepwise approach.

**Table 1:** Key methodological steps in calculation of burden of disease

Years of life lost to premature mortality	Years of life lived with disability	Disability Adjusted Life Year
Mortality data and definition of ill-defined deaths	Definition of disease	Age weighting and time discounting
Redistribution of ‘garbage codes’/ill-defined deaths	Counting disease frequency	Dealing with uncertainty in estimates
Choice of life table	Usage of disability weights	Choice of standard population
	Application of severity distributions	
	Multimorbidity corrections	

In highlighting the methogological challeges and decisions required in undertaking a BoD study as well as providing examples from individual countries in how they have addressed these challenges, this paper will identify the key challenges with the aim of working towards a joint BoD research agenda. This includes an identification of common challenges as well as knowledge and data gap, harmonizing methods and making BoD estimates more accessible to policy-makers.

## Estimation of YLLs

One component of the DALY metric is the measure of premature mortality or YLL. It can also be used as a standalone measure. The concept of YLLs is to estimate the additional length of time a person is expected to have lived, had they not died prematurely of a certain disease or injury. The YLLs are based on relating the age of death to an external standard life expectancy curve, and can incorporate time discounting and age weighting [14]. Furthermore, YLLs can be calculated for specific causes of death. In this way the indicator can be used to compare the relative importance of different causes of death within a particular population [15]. Thus, it can be used by health planners to define priorities for (preventive) interventions. The calculation of the YLLs is straightforward and incorporates the multiplication of the numbers of death in a reference year for a specific cause of disease or injury (*d*) with the remaining life expectancy at age of death (*l*) within each age (group) and sex category (*i*).

$$ YLL={\sum}_{i=1}^n{d}_i\ast {l}_i $$

**Table:** 

where: *i* = each age (group) from 1 to n *d* = number of deaths in each age (group) *i* *l* = standard life expectancy at age of death *i* (in years)	

However, the process of calculating the YLLs involves several data processing steps which include preparing the cause of death statistics in a form that can be used for the calculations (e.g. assessing the completeness of death reporting), and cleaning the data (e.g. redistribution of garbage codes). Some of the most significant steps will be described in the following section.

### Quality of mortality data and definition of ill-defined deaths

The main prerequisite for estimating YLLs is the availability of high quality mortality data. Usually countries with functioning vital registration systems have data with good quality and high population coverage. However, countries that do not have good vital registration systems need to perform some further efforts for obtaining mortality data. The World Health Organization (WHO) recommends the following steps [16]:

1. collation of all available data sources: health surveys, hospital discharges, medical registries, police records, etc.;
2. definition of a list of diseases (number of diseases that has to be analyzed in detail); and
3. following the list of selected diseases, estimation of mortality rates by cause, age and sex

For countries with vital registration systems, cause of death statistics coded using the WHO International Statistical Classification of Diseases and Related Health Problems (ICD) [17], contain valuable information on mortality in the general population. These statistics can be used in public health policy to identify the most important causes of death and to develop interventions and prevention policies. However, the cause of death statistics may contain mistakes made during data generation or have inaccurately coded deaths. Furthermore, the ICD classification system contains unknown or imprecise causes of deaths, which are termed as garbage codes in the GBD study. Others have preferred to describe such deaths as ‘ill-defined’ [18]. Such ill-defined deaths (IDDs) can include, for example, ICD codes that are not possible in certain age groups or sexes (for instance Alzheimer disease in infant ages, or testicular cancer in women); inaccurately coded deaths which may involve ICD codes that are not informative enough for public health policies (for instance unspecified cancer type). In many cases such IDDs may have been unavoidable as the underlying cause of death was not known to the medical person who diagnosed the death as it is not always possible to undertake extensive investigation to establish an exact cause of death [19].

The percentage of IDDs in the different countries can vary considerably: a recent study showed that for six high-income countries the IDDs varied from 22 to 36% [20]. Studies in Poland and South Korea have reported over a quarter of the cases in the cause of death statistics were IDDs [21, 22]. In contrast, in other countries, such as the Netherlands and Scotland, IDDs are approximately 10% of all causes of death [18, 23].

As in the context of BoD studies, all deaths must be assigned to one specific (and valid) cause of death, it is necessary to redistribute these IDDs accordingly. Alternatively, instead of a redistribution, the IDDs may be gathered in a rest category group [24]. However, most of the BoD studies opt for a redistribution of the IDDs. How this is done depends much on the countries’ death registration system and the information it collects on the underlying cause of death and other contributory factors/diseases. According to the availability of such information, different methods of redistribution of IDDs can be used.

### Methods of redistribution of ill-defined deaths

IHME uses a very sophisticated and comprehensive algorithm that defines a set of IDD types for the GBD, each of which is redistributed to a number of meaningful causes within each age and/or gender strata [25]. Where the cause of death is ill-defined the process aims to estimate the most probable cause of death based on the literature, expert opinion, ICD rules, and knowledge about the distribution of diseases in a country. Methods used for this redistribution include regression models, fixed proportions, proportional reassignment, and fractional assignment of a death assigned to multiple causes [26]. Up until recently, the algorithm used in GBD was based exclusively on the underlying cause of death recorded in the death certificate, alongside age and gender of the deceased. However for GBD 2017, IHME developed an algorithm for redistribution of garbage codes based on multiple causes of death i.e. the underlying cause of death as well as intermediate and immediate causes in the death chain. This approach was implemented in redistribution for a few selected causes e.g. misassignment of deaths due to drug overdoses to unintentional poisoning [27].

Due to the complexity of GBD redistribution methodology and difficulties in replicating the approach, some national BoD studies have sought to deal with the redistribution of IDDs using the available cause of death data in their country [28, 29]. Mostly the development of country specific methodology is driven by the available data in the given country. As an example, in information Case example 1 is presented a case study from Australia.

**Table:** 

*Case example 1: Redistribution of Ill-defined deaths. A case study from Australia*	
The most recent Australian BoD Studies [11, 30], conducted by the Australian Institute of Health and Welfare (AIHW), have used Australian data to develop methods for the redistribution of deaths.	
The first method uses direct data on plausible alternative causes of death for the deaths identified for redistribution. It uses information obtained through data linkage studies, sourced from deaths coded independently by cancer registries, and available on coroner-certified deaths and is considered the best method to use when suitable data are available.	
The second method (termed the Indirect multiple causes of death (MCoD) method) uses algorithms based on Australia’s multiple cause of death statistics. It is used for the most commonly occurring causes of death (e.g. heart failure, septicaemia, pneumonitis and hypertension) where no direct data is available. This method uses the pattern of the underlying causes of death (UCoD) where the cause identified for redistribution was mentioned as an associated cause of death. The corresponding UCoDs and their proportional distribution provide the redistribution algorithm.	
The third method, which uses proportional redistribution to specified target cause(s), is only used when neither of the other methods described above are suitable. This method reassigns deaths across a range of target causes selected according to: the existing distribution of underlying cause of death within that disease group, expert advice, or the GBD redistribution algorithms if considered appropriate for Australia [11].	
Using the Australian redistribution methods, approximately 10% of deaths were identified for redistribution in 2010. This compares with 18% of Australian deaths that were identified using GBD algorithms. The difference is largely due to the cause list used: some of the causes of death that were redistributed in the GBD study were directly allocated to a specified cause in the Australian study [30]. The largest numbers of deaths gained by redistribution in the 2015 Australian study were for cardiovascular diseases (5081 more deaths, an increase of 11%), cancer (4956 more deaths, an increase of 11%) and endocrine disorders (1648 more deaths, an increase of 46% largely due to deaths coded to unspecified diabetes being reassigned to Type 1, Type 2 and Other diabetes) [11].	

### Choice of life table

After the redistribution procedure of the cause of death statistics, information on the exact number of deaths for each cause of death for each age group and sex (possibly by region) is available. The next step for calculating the YLLs is choosing an appropriate life expectancy. The life expectancy within a country can be extracted from the national life tables.

Life tables are the means of translating age-specific mortality estimates into estimates of YLLs [3]. Life tables, also referred to as mortality or actuarial tables, use estimates of mortality rates and population counts to estimate period or cohort life expectancies. The remaining life expectancy at defined ages or age categories is required for use in BoD studies to facilitate YLLs calculations. There are two distinct issues which may affect end estimates:

- age categorisation and
- the expected value of remaining life years assigned at age of death.

Across BoD studies, both age-categorised and single-year life table approaches have been widely utilised. This choice is largely dependent on the availability of source data, as granular individual-level data on deaths is required to make use of single-year life tables. The impact of this choice is likely to be insignificant on final estimates, if one assumes J-shaped mortality rate curves by age. However, it is likely to be larger the earlier the final open-ended age-group starts, as the cut-off will determine how the mortality rate exponentially increases.

The major issue which impacts on the YLLs calculated is the value of remaining life years which is assigned at age of death. This has a significant impact on burden estimates and has important ethical distinctions [31]. There are three main methods which can be used to determine the value of remaining life years at a given age of death:

- Use of aspirational life table
- Use of observed or national life tables
- Using a fixed value

The first method is the use of an aspirational life table, such as used in the GBD study, which is referred to as the ideal standard [3]. Many other national studies, such as those in Australia, New Zealand and Turkey [32–34] have adopted this approach. A second approach is to use observed, or national life tables, which can be calculated from source data and are representative of the populations for which estimates are being reported. Countries such as Estonia, Austria, and Scotland have utilised this approach in their national studies [30, 35, 36]. Between these two approaches, evidence has illustrated the impact of how rates of DALYs and ranks of causes are affected. This choice directly affects deaths and has large implications for how we value YLLs in relation to YLDs [37]. Aspirational or standard life tables are a good way to facilitate comparisons between countries, as they perform a similar function as to when rates are standardised. Comparisons between observed, or national, life tables are difficult to be made between countries and even across time because no standard has been developed. The criticism of aspirational life tables is that they are not pragmatic, which is what observed life tables are seen to represent. Although there may be ethical implications of using stratified life tables that give different values of remaining life to different subgroups. These considerations are less of an issue if the study is cross-sectional in nature and seeks to describe population health loss without considerations of comparisons over time and across locations. From a methodological perspective, a standard must be set to enable comparisons and aspirational life tables are beneficial in this regard because there are always a subset of preventable deaths and improvement can always be strived towards [15].

The third and final approach is to determine the remaining life years at age of death based on a fixed value. This is a former method usually referred to as years of potential life lost (PYLL), which would assign a fixed value, such as 75 years, and determine the number of years lost based on the difference between 75 and the age of death with any deaths beyond age of 75 being assigned to the value of 0. The fixed value is most commonly set at a value which would be desirable, or realistic, for people to live to or an age below which mortality is considered preventable but ultimately the choice of a fixed value does remain arbitrary. Although this approach could be combined with an aspirational approach to suggest that people could live to older ages, such as the oldest reported death which occurred at 122 years of age [38].

Within the COST Action European Burden of Disease network [12] there is a consideration to establish a standard European life table where the highest observed life expectancy for each age group across all European countries is included. In this way comparisons between those countries can be easily conducted and there will be higher acceptance on national levels compared to the global aspiration life table.

## Estimation of YLDs

The other component of the DALY metric is the YLD, which measures the healthy time that is lost because of living with a disease or injury. YLDs are calculated by multiplying the prevalence or incidence of a disease by the short- or long-term loss of health associated with that disability (the disability weight), the disease duration and disease severity (severity distribution), respectively.

YLDs is calculated using one of the following formulae:

Incidence approach:

$$ YLD=I\ast DW\ast L $$

**Table:** 

Where: I is the number of incident cases in the reference period, DW is the disability weight (in the range 0–1), L is the average duration of disability (measured in years).	

Prevalence approach:

$$ YLD=P\ast DW\ast S $$

**Table:** 

Where: P is the number of prevalent cases in the reference period, DW is the disability weight (in the range 0–1), S is the severity proportion.	

The process of calculating a YLD involves several components and requires extensive epidemiological modelling and is often based on a diverse range of data sources, literature research, and/or expert opinion. In this section we will describe some of the key methodological challenges in calculating YLDs including the choice of data sources to estimate disease frequency, disability weights, severity distributions and adjusting for multimorbidities.

### Epidemiological estimates

Unlike cause of death information which largely relies on one data source, estimating YLDs will depend on a wide range of different data sources specific to each disease. It requires judgment on what the most plausible source of information is, or how different sources could be combined, and which parameters best describe the disability caused by each disease [16], Table 2.

**Table 2:** Key steps in describing disease epidemiology for burden of disease studies [16]

• What is the current knowledge on the disease being studied?	
• What are the limitations of the current knowledge on the disease being studied?	
• What are the relevant data available on the natural history of the disease and its disabling sequelae? (prevalence, incidence, duration, age of onset, remission rate, and mortality rate, level of severity and duration from disease onset to disabling sequela)?	
• If there are no precise data, is there at least a general consensus amongst disease experts?	

Unlike mortality data, there is often no single comprehensive and reliable source of data on incidence, prevalence, severity and duration of all non-fatal health conditions. Ideally, one would like to have data from a nationally representative system that continuously monitors the occurrence of all disorders in the population, based on a set of clearly defined diagnostic criteria. Instead, disease and injury morbidity estimates tend to be drawn from a wide variety of sources relying on what is available to describe the disease epidemiology. Preferably, disease estimates should be based on the best sources available, and should have case definitions appropriate to the disease being analysed [16, 28, 39]. Ideally, the necessary data should be collected through a systematic review of peer-reviewed literature and various sources of grey literature, including government agencies, non-governmental organizations and academia. Examples of the range of data sources that could be explored are provided in Table 3.

**Table 3:** Possible data sources

• Hospital statistics, including inpatient, outpatient attendances emergency department attendances	
• Maternity, birth records and Neonatal Care	
• Primary care data	
• Disease registers	
• Prescriptions	
• Population level health surveys	
• Communicable disease surveillance	
• Published literature	
• Grey literature, including government agencies, non-governmental organizations and academia.	

Administrative data sources (for example, disease registers, hospitalisations) can be evaluated for their level of ascertainment and coverage. Surveys can be assessed for their representativeness, potential selection bias and measurement bias (validity and reliability of measurement). Epidemiological studies should be evaluated for the quality of their study design, their timeliness, credibility, representativeness and sources of bias or error. All potential data sources (whether published or unpublished) should be assessed for their comparability, relevance and representativeness, currency, accuracy, validation, credibility and accessibility/ timeliness [39].

The availability of suitable data sources will often determine the scope of the BoD study. Countries embarking on a comprehensive BoD will face problems with data gaps. Some diseases are easier to cover than others based on data available in specific countries. There are few examples of countries who have conducted a full national BoD study covering a comprehensive range of conditions (e.g. Australia, the Netherlands) [40, 41]. A review of BoD studies carried out in the European Union found that 85% (169/198) of studies covered in review looked at a small range of diseases or just risk factors and often for specific research purposes only [8]. For instance, the Serbian BoD study includes 18 selected diseases and 7 risk factors [42], the Scottish BoD investigates 132 diseases and injuries [30] and the Netherlands’ study covers 101 diseases and injuries [43]. Furthermore, studies concentrating only on one or few diseases are also performed frequently [39, 44]. Other studies provide more detailed estimates for subgroup populations of interest. For instance, the Australian BoD study produces estimates for states and territories, by remoteness areas, by socio-economic group and for Indigenous and non-Indigenous Australians [32].

To date, the two most comprehensive sources of morbidity estimates for many countries are the most recent GBD studies conducted by IHME and the WHO. The GBD study provides a standardised approach for estimating incidence, prevalence, and YLDs by cause, age, sex, year, and location [25], where the methods used are continuously improved with each iteration. The study aims to use all accessible information on disease occurrence, natural history, and severity that passes a set of inclusion criteria. In addition to data sources based on primary literature, surveys, and surveillance, the GBD study has used an increasing number of hospital discharge records, outpatient visit records, and health insurance claims to inform various steps of the non-fatal modelling process.

The GBD study provides summary of estimates for all regions and countries of the world from a viewpoint of global average. However, data sources that are fed into the modelling process for country-level estimates can vary based on locations with limited availability of data [45–47]. This can lead to a high reliance on GBD to fill data gaps for these locations. The usage of very complicated calculation procedures in the GBD hampers the replication of the methodology on a country level [41, 48]. All this can limit the use of BoD estimates on a national level because even though international data comparisons provide a basis for policy discussion, policy makers often require assurance that these estimates are reliable in the local situation [5]. To address this and also produce a comprehensive national BoD study, researchers are using the GBD framework but adjusting certain methodological aspects to tailor it to the needs of the specific country using counry specific sources e.g. use of country specific disease classification, use of disease prevalence data derived from administrative patient databases instead of survey-based disease incidence estimation and use of national disability weights in accordance with the contextualized disease classification [37, 45, 49–51].

### Usage of disability weights

The disability weight is an essential factor to assess the YLDs because it translates morbidity into a theoretical survival loss by weighting survival for the time lived with functional capacity [52]. A disability weight reflects the impact of a disease or injury on a person’s life. Its value is anchored between 0 (equivalent to “full health”) and 1 (equivalent to “death”) and is commonly based on the health state valuations of a group of individuals [1]. Several sets of disability weights exist, such as the sets of GBD disability weights [3, 53, 54] as well as national sets [40, 55–58]. The set of GBD disability weights is used most frequently.

The first large set of global disability weights was derived for the GBD 1996 study. These disability weights were derived in a group exercise in which a panel of ten health experts evaluated 483 health states [3]. The choice to elicit global disability weights based on health state valuations of a small group of health experts was subsequently criticized [59, 60]. In the latest revisions of the GBD study, disability weights have been based on the health state valuations of over 60,000 people from the general public of a large number of countries to ensure that the disability weights reflected the views of the global population [54].

Apart from the characteristics of the individuals who provide the health state valuations, the value of the disability weight also depends on the description of the health state and the valuation methods that are used [61]. Health states can be described in generic terms or in disease-specific terms. A disease-specific description depicts the disease label and/or clinical description of the condition. A generic health state description depicts the functional health independent of the actual underlying condition. Table 4 provides examples of a disease specific and generic description of moderate to severe depression. Previous studies have shown that disease-specific health state descriptions provide information that is not reflected in the generic health states but that matters for health state valuation [63, 64]. On the other hand, disease specific health state descriptions may produce information bias because of message-framing effects [65, 66]. For the latest revisions of the GBD study, health states were described in both generic and disease specific terms, but without providing the disease label. An example of a health state description of the 2013 revision of the GBD disability weights is “this person has constant sadness and has lost interest in usual activities. The person has some difficulty in daily life, sleeps badly, has trouble concentrating, and sometimes thinks about harming himself (or herself)” (Major depressive disorder: moderate episode) [56].

**Table 4:** Example of a disease specific and generic description of moderate to severe depression, based on Kruijshaar et al. 2005 [62]

Disease specific description	Generic description
This person experiences one or more depressive episodes within a year. During these periods they go through permanent feelings of sadness or emptiness and a permanent loss of interest or pleasure in nearly all activities. He/she has problems eating and/or sleeping and feel worthless or guilty. He/she may have thoughts of death.	This person has some problems with performing usual activities, feeling tired, moderate anxiety or depression, some cognitive impairments.

The valuation methods that have been used to derive disability weights are the visual analogue scale (VAS), time trade-off (TTO), person trade-off (PTO) and paired comparison [61]. The VAS valuation technique requires participants to score the injury stage on a vertical scale graded from 0 (worst imaginable health state) to 100 (best imaginable health state). With TTO, the participants are asked how much time they would be willing to “trade” in order to be restored from the presented disease stage to full health. The person trade-off asks the participant how many outcomes of one kind (e.g. moderate to severe depression) they consider equivalent in social value to a set number of outcomes of another kind. With paired comparisons two descriptions of hypothetical health states are presented to respondents who have to decide which they regard as being healthier. These valuation methods give information about the relative desirability of a health state compared to other health states; however, the properties of the valuation methods affect the preferences that are measured. For instance, many studies have found that health state valuations with the VAS tend to be higher compared to equivalent valuations with choice-based valuation methods, such as the TTO and PTO [61, 67, 68]. For the latest revisions of the GBD study, paired comparison was used to elicit preferences for health states as well as population health equivalence (PHE) questions [54]. PHE questions ask for a retrospective assessment that compares two hypothetical health programs. Responses on population health equivalence questions were used to locate the health states on a 0–1 disability weight scale.

### Severity distributions

Estimates of the frequency of morbidity in a population, such as prevalence, are transformed into YLDs using disability weights for each disease-specific sequela. Severity distributions are a means of summarising the range of health loss suffered due to a disease which enables estimates of disease occurrence to be paired with disability weights, to estimate YLDs in BoD studies [69]. These distributions are usually expressed as the proportion of cases living with either: mild, moderate, severe, or no health loss (asymptomatic).

The GBD study applies the same severity distributions to all countries and regions across the world, which are largely based on data from three population surveys in the United States and Australia: the US Medical Expenditure Panel Survey (MEPS) 2000–2014, the [US] National Epidemiological Survey on Alcohol and Related Conditions (NESARC) 2000–2001 and 2004–2005, and the Australian National Survey of Mental Health and Wellbeing of Adults (NSMHWB) 1997. Each dataset containes individual-level measurements of functional health status as well as diagnostic information on the conditions affecting each individual [70]. The GBD study acknowledges concerns over applying estimates of severity distributions based on data from the United States and Australia, noting that it is the only available information that they were able to use because of inadequate data on severity from surveys or the epidemiological literature [69, 71].

Users of GBD estimates are, therefore, using an assumption of fixed severity distributions across populations. Researchers from independent national studies have been left with either: using the same approach as the GBD study; or developing their own country-specific severity distributions for all, or a subset of, causes. Pivotal examples of this are found in South Korea, Germany and Scotland, where researchers have opted to develop country-specific severity distributions [49, 72, 73]. A recent study highlighted a potential bias in point estimates of weighted-average disability weights created using worldwide cancer severity distributions [73]. This bias would have led to the misrepresentation of non-fatal (i.e. YLD) estimates of the burden of individual cancers, and underestimation in the scale of socioeconomic inequality in this non-fatal burden.

These issues raise uncertainties over interpreting YLD estimates, particularly if they are being used to develop and influence policies and to determine priorities across diseases and populations. It is clear that GBD researchers and those carrying out national studies need to work towards ensuring that estimates are based upon country-specific data, and, if possible, that the impact of assumptions are fully tested and understood [74]. Assessing the leading causes of YLDs and differences between the highest and lowest health state disability weights can be used to identify priority diseases for which it would be most beneficial to further develop severity distributions and help understand the wider uncertainties over applicability that are currently unanswered.

In most cases the estimation of severity distributions involves complex methodology as data for such assessments are very limited data or not reliable. When the appropriate data are obtained, the estimatation of severity distributions can be straight forward. In Information Case example 2 is given one example from the German project BURDEN 2020.

**Table:** 

*Case example 2: Etimation of severity distribution for migraine and low back pain. A case study from Germany*	
Using claims data as a single database to estimate prevalence and severity distributions for diseases like migraine or low back pain can be problematic. As usually people do not necessarily seek help from a physician if they suffer from acute headache or experience acute episodes of low back pain, there is a certain underreporting in terms of prevalence of these diseases.	
The German project BURDEN 2020, for instance, has conducted an own survey on migraine, low back pain and neck pain in order to operationalize the indicators as defined by the GBD study [75]. The survey enables to report estimates on the prevalence and severity distributions for each of these diseases. The results have shown that there are some differences in the country specific severity distribution compared to the GBD one, even if the same health states are measured. Furthermore, such an additional study provides the possibility to derive age and sex specific severity distributions which even further refine the estimated YLDs.	

### Multimorbidity corrections

With an ageing population, the prevalence of multimorbidity increases. Ignoring multimorbidity (i.e. co-occurrence of multiple diseases within one person [76]) results in a possible overestimation of the YLDs and thus of the overall disease burden [77, 78]. When no correction for multi-morbidity in an multi-cause BoD study is performed, then it automatically means that the disability weights of the comorbid diseases are added up [79]. This means that the additional effect on disability of one comorbid disease simply adds to the effect of the primary disease observed in uni-conditional patients. This is usually refered to as the additive approach.

To account for multimorbidity, estimates are required of the prevalence and the severity of combination of (two or more) diseases. There are several methods to calculate prevalence of multimorbidity and the combined disability weights. A systematic comparison of three different comorbidity adjustment approaches in patients with injuries and common diseases with non-trivial health impacts as the secondary condition was conducted by Haagsma et al. (2011) [79].

The most applied method in BoD studies is to assume independent prevalence and use a multiplicative model for combined disability weights [80, 81]. For persons with several health conditions, the simple additive approach may be problematic because adding disability weights on the individual level might lead to an overall disability above 1. A disability weight above 1 would imply that a year lived with disability is weighted higher than a year lost to death. To avoid this, the multiplicative method achieves a convergence of combined disability weights towards one [78]. To realize a data frame on an individual level, a simulated population is created. In order to reach the same disease prevalence as the original population subgroup of interest, diseases are independently assigned to simulants by assuming disease prevalence to be probabilities. The assumption of independence ignores the coexistence of and thus correlations between diseases. Then the disease burden of each individual in the simulated population is estimated using the formula:

$$ \mathrm{burden}\ \mathrm{for}\ \mathrm{individual}=1-\prod \limits_{\mathrm{d}=\mathrm{diseases}\ \mathrm{s}/\mathrm{he}\ \mathrm{s}\mathrm{uffers}\ }\left(1-\mathrm{disability}\_{\mathrm{weight}}_{\mathrm{d}}\right) $$

The burden for each simulated individual is later used to calculate a disability weight that is redistributed for all the diseases that affect a population subgroup [82]. This approach leads to a downward correction of around 10% for 25 chronic conditions. In reality, the assumption of independent occurrence might be a further overestimation, since different diseases have often shared risk factors (e.g. smoking relates to COPD and lung cancer), or certain diseases increase the risk for other diseases (diabetes and cardiovascular diseases).

As the multimorbidity adjustment is a complex issue and highly depends on the number of diseases under study, many countries have adapted the GBD approach to perform such corrections in the national studies [11, 83]. In information Case example 3 is described the approach used in the Netherlands.

**Table:** 

*Case example 3: Multimorbidity correction. A case study from The Netherlands*	
In the Netherlands, an adapted approach is used, where multimorbidity corrections are performed according to an independently-assumed occurrence of multiple disease data. This occurrence is calculated by gender, 5 year age groups, and the selection of long-term and chronic diseases. Combinations of up to five different diseases are considered. The multiplicative approach is applied for the disability weight of multimorbidity combinations [78].	

## The Estimation of DALYs

The DALYs are simple aggregation of the estimated YLDs and YLLs:

$$ \mathrm{DALY}=\mathrm{YLD}+\mathrm{YLL} $$

As mentioned above, DALYs quantify the health gap between a life lived in perfect health and current health status.

### Social weighting: age weighting and time discounting

In early GBD studies, two additional social weights, discounting and age weighting, applied to the calculation of the final YLLs [14]. In the GBD 2010 study, however, these social weighting functions were dropped, in order to simplify the calculation and interpretation of DALYs [6].

Age weighting implies that the value of life depends on age, such that greater weights are assigned to deaths at younger ages and lower weights to deaths at older ages. Age weighting may be used to increase or decrease the DALYs contributed by various age groups within a population if some age groups are societally deemed more “valuable” than others [84]. In early GBD studies, the weighting peaked at around 25 years, and decreased as age increased [14]. Discounting is an economic concept that applies higher weights to benefits that arise in the present relative to the future [3]. With discounting, an intervention that prevents 1000 cases of heart disease this year will remove more DALYs from a population’s total count of DALYs than an intervention expected to prevent 1000 cases of heart diseases from occurring 30 years from now. In the equations used to estimate DALYs, discounting assigns greater value to YLL reductions in the present than to years of life gained in future years [84]. GBD studies from the 1990s and early 2000s generally recommended using a 3% discount rate [14].

The use of age weighting and discounting in earlier GBD studies has been controversially discussed. Many of the assumptions and value judgements implicit in the choice of either social weighting were criticised [2, 85, 86]. Subsequently from 2010 onwards, discounting and age weighting were no longer used in GBD estimates [6, 87, 88]. Age weighting and time-based discounting, however, are still commonly used in national BoD studies [8, 9, 89, 90]. This can lead to considerable variability in the estimates of BoD depending on whether age weighing and discounting are used individually or together which have led to calls for transparency regarding the type of metric used and for a generally acceptable method that incorporates all the relevant social values to be developed [91].

### Dealing with uncertainty

Each estimate cannot be fully accurate and precise and carries with itself a kind of uncertainty that can have different sources. Normally, estimates are reported with confidence intervals or uncertainty bounds to account for possible inaccuracy of the estimates. Uncertainties can come from the data source but also can be produced by the modelling procedures and assumptions made [92]. As the GBD estimations involve very complicated methods or use data that is sometimes limited in its information, GBD publishes all YLLs, YLDs and DALYs estimates with uncertainty intervals which attempt to capture the random and systematic error in disease estimates.

Uncertainty bounds for all estimates in the GBD are assessed on each step of the modelling processes and involve a very complex estimation procedure. The GBD approach to describing and estimating uncertainty has been to define them as probability distributions using a Bayesian interpretation of probability as expressing uncertainty of an observed or hypothetical event given a set of assumptions about the world [93]. In GBD 2017, every estimate was calculated 1000 times, each time sampling from distributions rather than point estimates for data inputs, data transformations and model choice. The 95% uncertainty interval is determined by the 25th and 75th value of the 1000 values after ordering them from smallest to largest [71].

Deriving uncertainty in estimated disease burden is, however, difficult to do, because apart from the large number and disparate nature of the data sources used, information or knowledge about the quality of and potential biases in the data are often limited. Larger uncertainty intervals can result from limited data availability, small studies, and conflicting data, while smaller uncertainty intervals can result from extensive data availability, large studies, and data that are consistent across sources. Furthermore, the methods applied for generating uncertainty intervals in the GBD are difficult to replicate in other BoD studies. This has led to the development of own methods of uncertainty estimation [11, 30]. Example from Scotland is described in information Case example 4.

**Table:** 

*Case example 4: Dealing with uncertainty. A case study from Scotland*	
In order to provide a measure of the degree of accuracy and relevance of the estimated disease DALYs in the SBoD Study, a measure of data quality was developed by researchers, similar to that implemented in Australia [11, 94]. This measure assigns a RAG (Red; Amber; Green) status to each disease or injury indicative of the accuracy and relevance of the estimates [94].	
The data quality was assessed using the criteria below, on a scale of 1 to 5, with the weighted scores being assessed on a continuous scale:Morbidity (YLDs and prevalence):	
• Relevance and accuracy of the data source used to measure the population of interest;	
• Degree of adjustments performed to the input data;	
• Likelihood that the implemented disease model captured the burden of morbidity based on review of other data on disease prevalence in Scotland and the United Kingdom and disease expert advice/guidance.	
Mortality (YLLs and deaths):	
• Contribution of IDDs as a total of all deaths.	
DALYs:	
• Weighted-average of morbidity and mortality scores, where the weights were defined as the proportions of YLDs and YLLs of DALYs for the given cause of disease or injury for all ages and both sexes.	
These criteria are subjectively assessed and each criterion is scored on a scale of 1 to 5. Interpretation of the assigned RAG status is defined as: Green - highly accurate and relevant: Estimates have been derived using relevant and robust data sources with only a small degree of adjustments performed to the input data; Amber - Moderately accurate and relevant: Estimates have been derived using reasonably relevant and robust data sources with only a moderate degree of adjustments performed to the input data; and Red - uncertainties over accuracy and relevance: Estimates have been derived using less comprehensive or relevant data sources with a high degree of adjustments performed to the input data.	

### Impact from the choice of standard population

The final results of the YLLs, YLDs and DALYs calculation are usually presented not only in pure numbers, but also as rates. Rates are calculated because they frame the frequency of health loss in the perspective of defined populations. There are two main methods of rate calculations widely used in BoD studies: crude; and directly standardised, both of which have important uses and distinctions.

Crude rates should primarily be used to establish within-area prioritisation, or for when assessing the relative contribution of sub-groups across a larger region. Rates can also be used for monitoring across countries and temporal changes. Directly age-standardised rates (ASR) are required to facilitate this. ASRs use common reference population age-structure weights to enable the creation of artificial rates representative of a hypothetical scenario that would have occurred if the groups being compared had the same age distribution.

As the GBD study has an international remit, their ASR estimates are standardised to a world standard population (WSP). The WHO WSP was used in the first study iteration [3], with more recent GBD cycles using a GBD WSP in ASR calculations (GBD 2017). Other options to standardise rates are available, such as using the 2013 European Standard Population [93].

The Eurostat task force’s revision of the 2013 ESP highlighted that the plausibility and validity of ASRs come into question when residential populations have excessively different age structures than proposed standard populations [95]. Recent evidence focusing on differences between ASRs constructed using the 2013 ESP and GBD WSP illustrated that they were not only different in scale, but due to the effects of differences in age-weightings between standard populations there were significant changes in the rank order of causes [37]. Since the GBD WSP is a younger population than the 2013 ESP, causes that operate early in the life course would be expected to see relative gains, with those causes operating later in the life course observing reductions in ASR. Temporal effects in changing of the ranking of cardiovascular mortality across Europe have also been previously reported [96], highlighting that these issues are not only prevalent when assessing different causes of disease, but also across locations over time. This evidence means that there may be significant issues in knowledge translation for national BoD studies to consider, when crude and ASR estimates become excessively different.

## Summary and future developments

BoD studies provide a unique perspective on health, one that integrates fatal and nonfatal outcomes, yet also allows the two classes of outcomes to be examined separately. In addition, BoD analyses provides invaluable information that will assist in taking up the future challenges posed by an aging population, by changes in disease and risk factor patterns, and by the increasing costs of health services.

Driven by the impact of the GBD study, many countries have and initiated BoD studies for specific causes and/or geographies [8, 9]. The increasing prominence of the BoD approach, however, comes at a cost. The BoD methodology is complex and highly data demanding, requiring a substantial capacity to apply, which has led to major disparities across researchers and nations in their capacity to perform themselves BoD studies, interpret the soundness of available BoD estimates produced by IHME and others, or advocate for the use of BoD metrics.

In this review article we have identified some of the key methodological choices and challenges that are important to understand when calculating BoD metrics (YLLs, YLDs and DALYs). We have provided examples from different BoD studies that have developed their own strategies in data usage and implementation of statistical methods in the production of BoD estimates.

As more and more countries are implementing or using the BoD approach, there is an increasing need for equitable capacity building on the one hand (including an improved understanding of the complex methods behind IHME and other burden estimates), and harmonization of methods on the other hand. Furthermore, current evolutions in public health, including big data and precision public health, call for a technical platform to foster the integration of these concepts in the BoD approach. This problem is acknowledged by all researchers involved in BoD studies and it is increasingly recognised that there is a high need for knowledge transfer, capacity building and putting joint efforts together in improving the methodology and statistical modelling.

The COST Action European Burden of disease network has been established to provide a technical platform to integrate and strengthen capacity in BoD assessment across Europe and beyond [12]. One of the aims of the network is to provide a checklist and a road map for conducting BoD study which may allow a comparison between the national BoD studies in the future. Furthermore, an intensive exchange in the experience of the researchers in the different countries will enable the understanding of the methods and the interpretation of the results from the local authorities who can effectively integrate the BoD estimates in public health policies, intervention and prevention programs.

## Data Availability

Not aplicable.

## Abbreviations

ABDS: Australian Burden of Disease Study
AIHW: Australian Institute of Health and Welfare
ASR: Age-standardised rates
BoD: Burden of disease
DALYs: Disability-adjusted life years
ESP: European Standard Population
GBD: Global Burden of Disease
ICD-10: International Statistical Classification of Diseases and Related Health Problems - 10th revision
IDD: Ill-defined deaths
IHME: Institute for Health Metrics and Evaluation
InFact: Joint Action on Health Information
MCoD: Multiple causes of death
PHE: Population health equivalence
PTO: Person trade-off
PYLL: Potential years of life lost
SMPH: Summary measures of population health
TTO: Time trade-off
UCoD: Underlying causes of death
VAS: Visual analogue scale
WHO: World Health Organization
WSP: World standard population
YLDs: Years lived with disability
YLLs: Years of life lost
